# AI-Driven Clinical Decision Support to Reduce Hospital-Acquired Venous Thromboembolism: A Trial Protocol

**DOI:** 10.1001/jamanetworkopen.2025.35137

**Published:** 2025-10-03

**Authors:** Colin G. Walsh, Yufei Long, Laurie Lovett Novak, Megan E. Salwei, Benjamin Tillman, Benjamin French, Amanda S. Mixon, Michelle E. Law, Jacob Franklin, Peter J. Embi

**Affiliations:** 1 Department of Biomedical Informatics, Vanderbilt University Medical Center, Nashville, Tennessee; 2 Department of Medicine, Vanderbilt University Medical Center, Nashville, Tennessee; 3 Department of Psychiatry and Behavioral Sciences, Vanderbilt University Medical Center, Nashville, Tennessee; 4 Center for Research and Innovation in Systems Safety, Department of Anesthesiology, Vanderbilt University Medical Center, Nashville, Tennessee; 5 Division of Hematology and Oncology, Department of Medicine, Vanderbilt University Medical Center, Nashville, Tennessee; 6 Division of Laboratory Medicine, Department of Pathology, Microbiology, and Immunology, Vanderbilt University Medical Center, Nashville, Tennessee; 7 Department of Biostatistics, Vanderbilt University Medical Center, Nashville, Tennessee; 8 Department of Medicine, Vanderbilt Wilson County Hospital, Lebanon, Tennessee; 9 Department of Medicine, Vanderbilt Bedford County Hospital, Shelbyville, Tennessee; 10 Department of Medicine, Vanderbilt Tullahoma Harton Hospital, Tullahoma, Tennessee

## Abstract

**Importance:**

Hospital-acquired venous thromboembolism (HA-VTE) remains a leading cause of preventable death among hospitalized adults in the US. Despite numerous attempts to prognosticate HA-VTE with risk models, no single model has outperformed the rest, and the effectiveness of such models to drive prophylaxis decisions is unknown. Testing such systems in urban and rural settings may inform their generalizability.

**Objective:**

To conduct a randomized clinical trial to assess the effectiveness of artificial intelligence (AI)–driven clinical decision support (CDS) in reducing HA-VTE incidence in adults across urban and rural hospital settings.

**Design, Setting, and Participants:**

This parallel-group, single-blind, pragmatic randomized clinical trial is planned to be conducted from October 1, 2025, through September 30, 2027, by the Vanderbilt University Medical Center, a major academic health system in Tennessee. The study population will include adult (aged ≥18 years) patients admitted to medical, surgical, and intensive care units who may be at high risk for VTE and with no active or contraindication to deep vein thrombosis prophylaxis at Vanderbilt Adult Hospital in urban Nashville and 3 affiliated hospitals serving rural communities in Middle Tennessee.

**Intervention:**

Patients will be randomized 1:1 within the electronic health record to receive either VTE-AI–driven CDS (nudge practice alert [intervention arm]) or standard care using traditional risk assessment (control arm).

**Main Outcome and Measures:**

The primary outcome will be incidence of HA-VTE. Secondary trial outcomes will be process metrics, including length of stay, readmission rates, safety, and bleeding events. Outcomes will be analyzed using descriptive statistics and compared using Poisson regression.

**Discussion:**

Using a validated prognostic model, this study is one of the first to provide insights into whether AI-driven CDS can effectively reduce HA-VTE incidence without increasing adverse events. This study also is intended to provide insights into the usefulness of the same AI model implemented across urban and rural settings. The study’s findings and statistical code will be shared with the public through peer-reviewed publication and ClinicalTrials.gov.

**Trial Registration:**

ClinicalTrials.gov Identifier: NCT06939803

## Key Points

**Question:**

Does a prognostic model–driven electronic health record alert recommending venous thromboembolism (VTE) prophylaxis for hospitalized adults who may be at high risk, are not receiving active prophylaxis, and do not have a contraindication reduce the incidence of hospital-acquired VTE?

**Findings:**

This trial protocol describes the Venous Thromboembolism Using AI (VTE-AI) randomized clinical trial, which will test a prognostic model–driven electronic health record–based nudge to reduce hospital-acquired VTE incidence across urban and rural hospital settings in Tennessee.

**Meaning:**

The VTE-AI trial is one of the first randomized clinical trials to measure the effectiveness of health care AI–driven decision support compared with standard of care across medical and surgical services and urban and rural settings.

## Teaser

This trial protocol describes a randomized clinical trial to assess artificial intelligence (AI)–driven clinical decision support in reducing hospital-acquired venous thromboembolism incidence in hospitalized adults across urban and rural settings in Tennessee.

## Introduction

Hospital-acquired venous thromboembolism (HA-VTE) remains a leading cause of preventable death among hospitalized adults in the US.^1,2^ Approximately 900 000 people experience VTE each year, with incidence-based medical costs estimated between $7 and $10 billion per year.^3^ The second leading cause of disability-adjusted life-years, HA-VTE causes significant morbidity and mortality among adult and pediatric patients.^4^ Approximately 1 in 3 people experience long-term complications (ie, postthrombotic syndrome) following VTE. Reducing HA-VTE presents a major preventive challenge.

Despite numerous published prognostic models for HA-VTE, no single model has outperformed the rest, and HA-VTE affects groups inequitably, which means that models might worsen care if they are not deployed in the context of responsible, algorithmovigilant systems.^5^ Integrating scalable artificial intelligence (AI) for HA-VTE prevention into effective clinical decision support (CDS) might effectively reduce HA-VTE incidence while aiding the realization of the potential for AI in high-value clinical practice. Recently, a Vanderbilt University Medical Center (VUMC) team of clinicians and biostatisticians developed and validated a real-time prognostic model for HA-VTE called Venous Thromboembolism Using AI (VTE-AI) for hospital inpatients.^6^ The model includes medical history variables, vital signs, and laboratory measurements; can be updated as the clinical scenario evolves; and exhibits excellent prediction performance (C statistic, 0.891; 95% CI, 0.882-0.900; integrated calibration index, 0.001), without performance differences by age, sex, race and ethnicity, and type of admission.^6^ The VTE-AI model is undergoing local, recurring validation in preparation for this randomized clinical trial (RCT).^7^

The urban-rural divide has long caused health care disparities in morbidity and mortality.^8^ These differences might not result from rurality itself but from “the effects of socio-economic disadvantage, ethnicity, poorer service availability, higher levels of personal risk and more hazardous environmental, occupational and transportation conditions.”^9^^(p56)^ Artificial intelligence implementation may be no different without close attention to differences in both deployment settings. Studying multiple simultaneous implementations of AI in both urban and rural settings with adult and pediatric patients may yield unprecedented insights for AI-driven CDS.

The goal of this study is to evaluate the effectiveness of AI-driven CDS to reduce the incidence of HA-VTE across the Vanderbilt health system, including the following sites: Vanderbilt Adult Hospital (VUH) in urban Nashville, Tennessee, and the Vanderbilt Regional Health System in rural Tennessee, including Vanderbilt Tullahoma Harton Hospital (VTHH), Vanderbilt Bedford County Hospital (VBCH), and Vanderbilt Wilson County Hospital (VWCH). This trial intends to rigorously study a novel AI-based approach to estimating HA-VTE risk and guiding prevention. We hypothesized that this approach will produce strong evidence for the pragmatic use of novel AI-driven CDS to prevent HA-VTE across diverse sites and populations.

## Methods

This article was prepared in accordance with the Standard Protocol Items: Recommendations for Intervention Trials (SPIRIT) reporting guideline.^10^ The trial was approved by the VUMC Institutional Review Board, with waiver of informed consent given the impracticability of consenting every hospitalized patient at 4 hospitals and the potential introduction of bias via consent in the decision-making process to prescribe VTE prophylaxis. Data will not be deidentified, and the trial is considered minimal risk as VTE prophylaxis remains standard of care and the nudges prompted by this trial will be targeted only to patients who may be at high risk, are without contraindications as judged by the primary admitting team, and do not already have an order for VTE prophylaxis. Given the minimal risk and reliance on prompting a standard-of-care prophylaxis, no data safety monitoring board was convened for this RCT. The trial protocol is provided in Supplement 1.

### Study Design

The VTE-AI trial is a parallel-group, single-blind, pragmatic RCT planned to be conducted from October 1, 2025, through September 30, 2027, across all inpatient units at VUH, VTHH, VBCH, and VWCH. Study hospitals will enroll patients for 12 months. The standard of care includes order sets suggesting prophylactic options or documentation of a temporary or permanent exception or contraindication to prophylaxis. We will evaluate the effectiveness of the VTE-AI–driven CDS against the standard deep vein thrombosis and VTE prophylaxis workflows to reduce HA-VTE incidence across urban and rural dimensions. We will conduct a pragmatic RCT of VTE-AI–driven CDS that randomizes half of the eligible encounters to CDS and half to standard of care.

#### Population

The study will be conducted at VUMC, an academic medical center in the midsouth of the US. Within VUMC, the trial will be conducted in VUH, the main urban adult hospital in Nashville, and Vanderbilt Regional Health System, a set of regional hospitals in Middle Tennessee. All adult patients aged 18 years or older being admitted for inpatient care to VUH, VTHH, VBCH, or VWCH will be eligible for VTE-AI calculation and subsequent randomization.

#### Intervention

We will implement a validated risk score, VTE-AI,^6^ which does not require manual clinician input to calculate, to prompt CDS suggesting reconsideration of VTE prophylaxis among patients who (1) do not have an active prophylaxis ordered and (2) have no contraindication to pharmacologic prophylaxis. The VTE-AI is a prognostic model for HA-VTE developed from 132 330 adult patient encounters from 2018 to 2020 at VUMC.^6^ The model uses 25 factors selected from an initial candidate list of 82 risk factors, including demographic and clinical characteristics, diagnostic procedures, vital signs, and laboratory measurements (eg, complete blood counts, chemistry panels). All risk factors are routinely collected and available for prognostic calculation in real time, such as insertion of a central venous catheter, history of cardiac arrhythmia, recent weight loss, admission source, and C-reactive protein. This model is a statistical risk model that uses logistic regression, which means that the aforementioned variables are used in a mathematical equation to estimate a probability that HA-VTE will occur for that individual in the future. As an initial implementation of the VTE-AI model in the EHR, we have selected a CDS nudge that will occur starting on the day after hospital admission orders have been written and on each subsequent day of an inpatient encounter unless prophylaxis is ordered or a contraindication documented.

#### EHR Nudge

**Figure 1.**
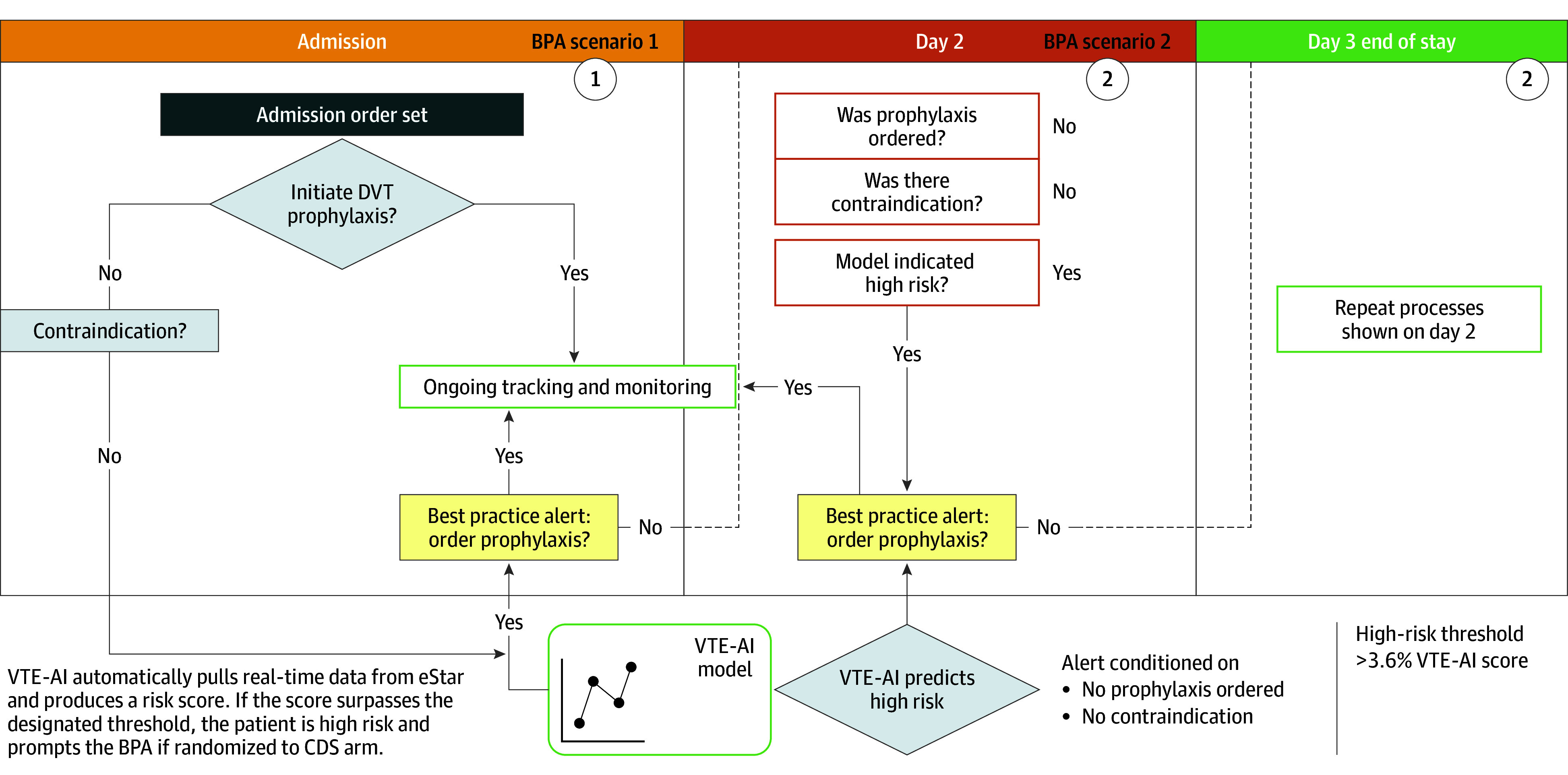
Intervention OurPractice Advisories Logic BPA indicates best practice advisory; CDS, clinical decision support; DVT, deep vein thrombosis; VTE-AI, Venous Thromboembolism Using Artificial Intelligence.

The CDS intervention will use the automated VTE-AI risk score to add EHR-based prompts in the form of OurPractice Advisories (OPAs) targeting those encounters for which (1) VTE-AI risk is above threshold (>3.6% estimated risk), (2) no active deep vein thrombosis prophylaxis pharmacologic order is present, and (3) no contraindication has been documented in the current aforementioned admission order sets. The schematic in Figure 1 shows the implementation logic.

**Figure 2.**
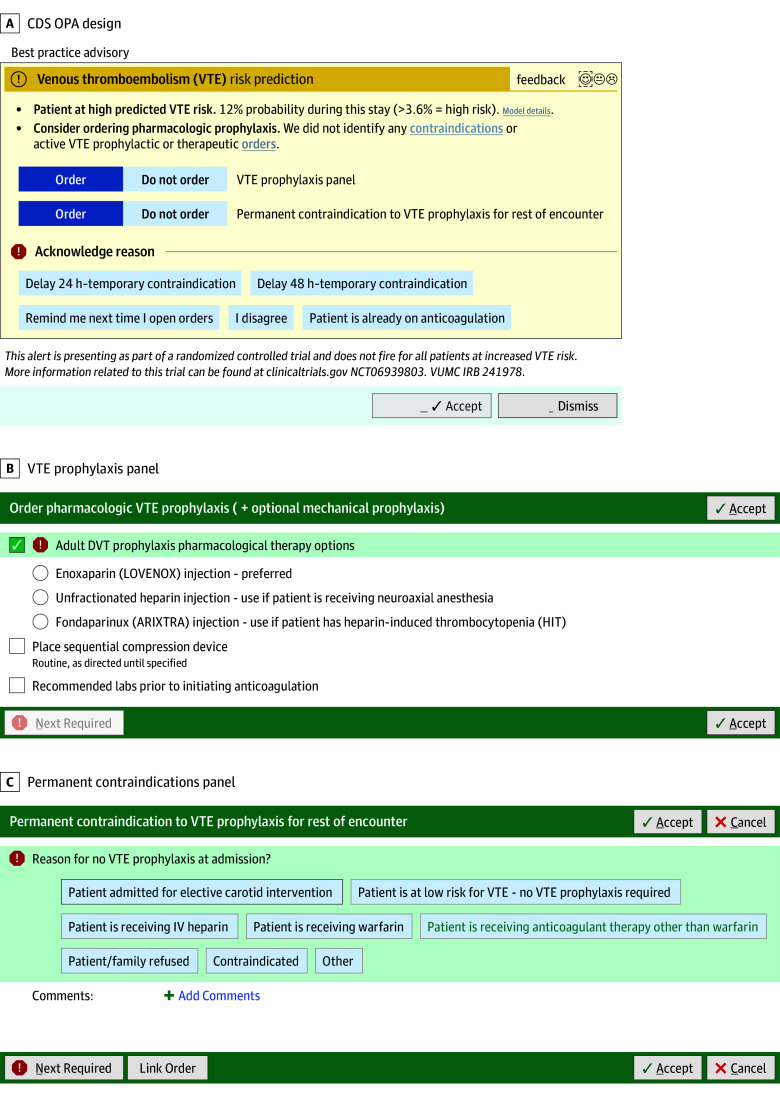
Venous Thromboembolism (VTE) Using Artificial Intelligence Electronic Health Record Nudge DVT indicates deep vein thrombosis; IV, intravenous; OPA, OurPractice Advisory.

The EHR nudge was designed using best practices in implementation science and human-centered design, including iterative design, heuristic evaluation, and premortem planning sessions, to foresee and prevent errors resulting from design issues. On opening the orders context in the EHR, the OPA will trigger each day of an inpatient encounter starting on hospital day 2. The CDS OPA (Figure 2) will prompt consideration of pharmacologic prophylaxis (VTE prophylaxis panel) and opportunities to take alternate actions, including disagreeing with the alert, entering temporary or permanent contraindications, or indicating that the patient is already receiving acceptable prophylaxis. Both the VTE prophylaxis and permanent contraindications panels are already deployed in medical units at VUH.

Both contraindications and medications deemed acceptable prophylaxis are listed in the eAppendix in Supplement 2. As examples, contraindications might be permanent (eg, history of heparin-induced thrombocytopenia, bleeding disorders) or temporary (eg, planned procedure, low platelet count <50/µL). The CDS will be discontinued by any action in the OPA or the recommended or alternate actions, except for the Dismiss and Temporary Contraindications actions (Figure 2).

#### Outcomes

The primary study outcome is occurrence of HA-VTE during hospitalization. The secondary outcomes are bleeding events and process measures of 30-day unplanned readmission and length of stay.

#### Randomization and Allocation

Admitted patients with VTE-AI scores above or equal to a risk threshold of 3.6%, the optimal F1-score threshold, will be randomized 1:1 to the intervention or comparator arm. The F1-score is a common metric to evaluate model performance at a given threshold and remains relevant here as HA-VTEs are rare events, so precision, also known as positive predictive value, is critical. The F1-score is calculated as the harmonic mean of positive predictive value and sensitivity (precision and recall, respectively, in information retrieval terms). Randomization will be conducted using a hashed value combining patient medical record number and admission date to ensure a reproducible trial arm assignment that will not change during each encounter from admission to discharge.

#### Concealment and Blinding

Because of the use of nudges in the RCT, concealment of the intervention will not be possible for clinicians making prophylaxis decisions. Patients will be masked to intervention status unless ordering clinicians share their decision-making rationale with them.

#### Safety End Points

The primary safety concern with prophylaxis remains bleeding events, and these will be monitored as a secondary trial outcome. Bleeding events will be identified after hospitalization using a published list shown to be sensitive.^11^ Each hospitalization in the intervention or comparator arm that evidences one of the referenced bleed-related diagnostic codes will then be manually reviewed by the EHR team in line with Joos et al^11^ who recommended the use of *International Statistical Classification of Diseases, Tenth Revision* codes to identify bleeding complications only with confirmatory EHR review.

### Statistical Analysis

We hypothesize that CDS will reduce the incidence of HA-VTE among patients (1) considered to be at high risk by VTE-AI and (2) without evidence of pharmacologic prophylaxis in half, from baseline 4.3% (562 of 12 946 events) incidence to 2.2% incidence, which will require 2236 encounters. Sample size calculation indicated that at least 1118 patient encounters are needed in each arm for a 2-sided binomial test of proportions to achieve 80% power with 5% probability of type I error. Using historical data from 2023 to 2024, we observed encounter counts of 150 to 230 per month that met these criteria. We will therefore conduct the RCT for 1 year to meet the required sample size.

Primary and secondary outcomes will be analyzed as counts and percentages of admissions. Between-arm comparisons will be conducted using Poisson regression to adjust for confounders and assess differences in incidence rates. For example, bleeding events (secondary outcome) might be affected both by the intervention itself and covariates, including exposure time to prophylaxis, age, clinical comorbidities, etc. Algorithmovigilance will be assessed by evaluating model validity to prognosticate HA-VTE in the comparator arm by key subgroups prone to potential unfairness, including age, race and ethnicity, and insurance status.^12^ Validity will be measured via calibration and model discrimination performance by subgroup.

#### Data Management

Data will be collected from the EHR and stored securely in the VUMC Data Center on servers owned by the study team for later analysis. These systems are designed to securely store Protected Health Information and include encrypted storage, multifactor authentication, and both physical (locked doors, secure facility) and technical (active directory user authentication) controls. Protected Health Information, while necessary to conduct study analyses, will never be exported from these servers or shared. Data to be collected to calculate VTE-AI include type of admission; heart rate; diagnostic codes (for clinical comorbidities); central line placement data; and laboratory data, including basic metabolic panel and C-reactive protein.

#### Dissemination of Trial Findings

Upon trial completion and data analysis, we will share the study’s findings via peer-reviewed publication and ClinicalTrials.gov. We will make the protocol and statistical code available to the public. Authorship will adhere to the standards set by the International Committee of Medical Journal Editors.

#### Data Sharing Plan

Because of the sensitive nature of the Protected Health Information needed to conduct this trial, public access to participant-level data will not be possible. Statistical code and the full protocol for the study will be disseminated with peer-reviewed publication, as appropriate.

## Discussion

The VTE-AI RCT is intended to inform multiple key questions in inpatient health care; prevention of patient safety events; and scalable, AI-driven informatics tools to guide care. The effectiveness of EHR nudges remains a topical question in the era of alert fatigue and the rise of health care AI requesting clinician attention. Given that VTE prophylaxis is standard of care, nudging admitting teams only for patients who may be at highest risk without documented contraindications might reduce the burden of nudges while improving quality of prophylaxis, as intended here. To our knowledge, behavioral nudges have been studied in the context of cardiology and oncology but not in hematology or in daily prophylaxis decisions at this scale.^13,14^

The health system scale intended for this RCT also may inform differences in risk enrichment and in care delivery across urban and rural practice settings. These differences might inform how interventions codesigned with admitting physicians could achieve effective CDS in a range of settings in which admitting workflows and admitting teams might vary. The generalizability of these findings might be enhanced by their application in medical, surgical, and intensive care settings, as well as by their application in both urban academic hospitals and rural community hospitals affiliated with a health system.

### Limitations

There are several potential limitations to validity and generalizability of the trial results, which include the focus on clinical sites in 1 region of a single state. The diversity of admitting workflows might result in disparate uptake of the AI-driven nudges across different settings. To mitigate this effect somewhat, the nudges will occur on subsequent days of hospitalization in gaps in which no competing workflows might provide conflicting guidance to clinicians. Another potential limitation relates to contamination. Admitting physicians exposed to CDS nudges might generalize their behavior to similar patients not receiving nudges, which might dilute effects of the intervention. We anticipate this effect will be limited by the focused integration of the CDS into existing workflows and lack of explicit information about patient eligibility in the comparator arm. Finally, risk models drift over time for various reasons, ranging from outcome drift to feature drift. This well-known phenomenon might affect model performance but will not be diagnosed or corrected in the scope of the RCT.

## Conclusions

The VTE-AI trial represents one of the first AI-driven decision support trials to reduce HA-VTE incidence, a potentially fatal patient safety event, to be conducted in a range of clinical and urban and rural settings. This trial has potential clinical implications in scalable approaches to prompt evidence-based practice while reducing alert fatigue through targeted nudging across large, heterogeneous clinical systems. It also might inform policy development incentivizing cross-site validation prior to widespread adoption of such AI-driven, quality improvement–oriented systems.

## Notes

Supplement 1.
Trial Protocol

Supplement 2.
eAppendix.

Supplement 3.
Data Sharing Statement
