# Supplementary material for: AI-Driven Clinical Decision Support to Reduce Hospital-Acquired Venous Thromboembolism: A Trial Protocol
Source: JAMA Netw Open. 2025 Oct 3;8(10):e2535137. doi: 10.1001/jamanetworkopen.2025.35137 (PMC12495493; doi:10.1001/jamanetworkopen.2025.35137)
Supplement: Supplement 1. — Trial Protocol [file jamanetwopen-e2535137-s001.pdf]

Preventing Hospital-Acquired- Venous Thromboembolism with AI-driven Clinical Decision Support: Study Protocol for the VTE-AI Trial

Colin G Walsh, MD, MA
2525 W End Ave, Suite 1475
Nashville, TN 37203
Department of Biomedical Informatics
Department of Medicine
Department of Psychiatry and Behavioral Sciences
Vanderbilt University Medical Center

colin.walsh@vumc.org

Trial Registration

ClinicalTrials.gov Identifer: Pending

URL: https://clinicaltrials.gov/study/[Pending]

Funding: AIM-HI, Kaiser Permanente and the Moore Foundation

Table of Contents:

Administrative Information
1.0 Roles and Responsibilities
2.0 Trial Sponsor
3.0 Role of study sponsor/funders
4.0 Composition of steering committee, data management teams

Study Schema
1.0 Background
2.0 Rationale and Specific Aims
2.1 Objectives
3.0 Animal Studies and Previous Human Studies
Methods
4.0 Inclusion/Exclusion Criteria and Setting
5.0 Enrollment/Randomization
6.0 Study Procedures
7.0 Risks of Investigational Agents/Devices (side effects)
8.0 Reporting of Adverse Events or Unanticipated Problems involving Risk to Participants or Others
9.0 Study Withdrawal/Discontinuation
10.0 Statistical Considerations
11.0 Privacy/Confidentiality Issues
12.0 Follow-up and Record Retention
13.0 Ethics and Dissemination
14.0 Appendices

Administrative Information

1.0 Roles and Responsibilities

Protocol contributors: Study Co-Principal Investigator, Colin Walsh, drafted, revised and received approval for the trial and its protocols. The protocol was reviewed by all Study Co-PIs: Dr. Peter Embi, Dr. Laurie Novak, Dr. Megan Salwei, and by all domain expert Study Co-Is: Dr. Benjamin Tillman, Dr. Ben French, Dr. Amanda Mixon.

Dr. Walsh is affiliated with Vanderbilt University Medical Center, the host organization and study location. The Study Coordinator, Katelyn Robinson, assisted in organizing, delegating, and managing ongoing trial activities.

2.0 Trial Sponsor: None

3.0 Role of study sponsor/funders: Funders played no role in design and conduct of the study; collection, management, analysis, and interpretation of the data; preparation, review, or approval of the protocol or related manuscripts.

4.0 Composition of steering committee, data management teams: The trial team managed trial data in partnership with Health Information Technology (HealthIT) at the trial location. The ongoing review of study data by the trial team ensured that the clinical study could continue without jeopardizing participant safety and the continuing validity and scientific merit of the trial. Because the study team had developed, validated, and analyzed the precise Electronic Health Record (EHR) data needed to conduct the trial, the team led on data and safety monitoring. The PI and study coordinator were responsible for monitoring the safety of participants, and for ensuring that participants are not exposed to undue risk.

Study Schema

1.0 Background

Hospital Acquired Venous Thromboembolism (HA-VTE) remains the leading cause of death in hospitalized patients in the US. Approximately 900,000 people experience VTE each year, with incidence-based medical costs estimated between $7 and $10 billion per year. The second leading cause of disability-adjusted life-years, HA-VTE causes significant morbidity and mortality in adult and pediatric patients. Roughly 1 in 3 people experience long-term complications (i.e., post-thrombotic syndrome) following VTE. Reducing HA-VTE presents a major diagnostic challenge.

Despite numerous published prognostic models of HA-VTE, no single model outperforms the rest. And HA-VTE affects groups inequitably, which means models might reflect or worsen healthcare disparities if they are not deployed in the context of responsible, algorithmovigilant systems. Integrating scalable AI for HA-VTE prevention into effective clinical decision support (CDS) might effectively reduce HA-VTE incidence while aiding the realization of the potential for AI in high-value clinical practice. Recently, a Vanderbilt team of clinicians and biostatisticians validated a regression risk score called "VTE-AI" to prognosticate risk of HA-VTE on admission (DOI: 10.1016/j.rpth.2024.102433).

The urban-rural divide has long caused healthcare disparities in morbidity and mortality. These differences might not result from rurality itself, but from "the effects of socio-economic disadvantage, ethnicity, poorer service availability, higher levels of personal risk and more hazardous environmental, occupational and transportation conditions."[37] AI implementation will be no different without close attention to differences in both deployment settings. Studying multiple simultaneous implementations of AI in both urban and rural setting with adult and pediatric patients will yield unprecedented insights for AI-driven CDS.

2.0 Rationale and Specific Aims

This study will rigorously study *novel AI approaches* to this *important diagnostic challenge* and produce strong evidence for the pragmatic use of novel AI-CDS to prevent HA-VTE across diverse sites and populations.

The goal of this study is to evaluate the effectiveness of AI-driven CDS to reduce the incidence of HA-VTE across two dimensions: urban (Vanderbilt Adult Hospital [VUH]) and rural (Vanderbilt Regional Health System [VRHS] including Vanderbilt Tullahoma Harton Hospital [VTHH], Vanderbilt Bedford County Hospital [VBCH], and Vanderbilt Wilson County Hospital [VWCH]).

We will implement a validated risk score, VTE-AI, which does not require clinician input to calculate, to prompt CDS suggesting reconsideration of DVT prophylaxis in those who 1) do not have active prophylaxis ordered and 2) have no contraindication to pharmacologic prophylaxis. This CDS "nudge" will occur after admission orders have been submitted for hospital admissions and on each subsequent day of an inpatient encounter.

The current standard of care is an order set requiring manual calculation of the Padua risk tool and selection of a prophylaxis option or documentation of a temporary or permanent exception. We will evaluate the effectiveness of the VTE-AI-driven clinical

decision support (CDS) against the standard DVT/VTE prophylaxis order set to reduce HA-VTE incidence across urban/rural dimensions.

Aim 1: Prospectively validate the VTE-AI risk score in clinical production systems. We hypothesize the VTE-AI risk score will be non-inferior in clinical production compared to the retrospectively validated model (benchmark c-statistic > 0.8)
Aim 2: Develop a modified, simplified version of the DVT/VTE order set that does not require manual calculation of the Padua risk tool (standard of care) and assess its acceptability with clinicians in trial sites.

Aim 3: Conduct pragmatic RCT of VTE-AI-driven CDS against standard of care at VUH and VTHH. We will implement CDS at VUMC and VTHH randomizing half the eligible encounters to CDS and half to standard of care.

2.1 Objectives

Primary Objective

We hypothesize CDS will reduce incidence of HAVTE in those i) predicted at high risk by VTEAI and ii) without evidence of pharmacologic prophylaxis in half, from baseline 4.3% incidence (562/12,946 events) to 2.2% incidence, which will require 2,236 encounters.

Secondary Objective

We hypothesize the VTE-AI CDS will not increase bleeding risk, readmission rates, or lengths of stay (LOS) between the intervention and non-intervention arms.

3.0 Animal Studies and Previous Human Studies

No animal or human studies have been done.

4.0 Inclusion/Exclusion Criteria and Study Setting

Patient Inclusion Criteria
-Inpatient admission to VUH or VRHS (standard of care is all admitted patient encounters include the DVT/VTE prophylaxis order set as part of admitting orders)

Patient Exclusion Criteria
-None

Study Setting

The study is conducted at Vanderbilt University Medical Center (VUMC), an academic medical center in the Mid-South of the United States. Within VUMC, the trial is conducted in VUH, the main urban adult hospital in Nashville, and VRHS, a collection of hospitals including a rural hospital in Tullahoma, Coffee County, TN.

5.0 Enrollment/Randomization

Randomization will be done in the Epic EHR, built-in functionality. One half will be randomized to VTE-AI-driven CDS and one-half to standard of care.

Eligibility Criteria

All patients being admitted for inpatient care to VUMC Adult Hospital, Vanderbilt Tullahoma Harton Hospital, Vanderbilt Bedford County Hospital, or Vanderbilt Williamson County Hospital during its trial period.

6.0 Study Procedures

Prediction with VTE-AI

VTE-AI uses a prognostic model of HA-VTE validated on 132,365 adult patient encounters from 2018-2020. The model uses 39 factors selected from a diverse candidate list of 84 risk factors ranging from demographic and clinical characteristics, diagnostic procedures, vital signs, and laboratory measurements (e.g., complete blood counts, chemistry panels). All predictors are routinely collected and available for prognostic calculation in real-time, such as insertion of a central venous catheter (c^2: 192.1), history of cardiac arrhythmia (c^2: 175.8), recent weight loss (c^2: 96.2), admission source (c^2: 77.8), and C-reactive protein (c^2: 43.1). This model has performed with good to excellent discriminative ability thus far (c-statistics 0.89-0.9).

This model is a statistical risk model using logistic regression, which means the variables above are used in a mathematical equation to estimate a probability that HA-VTE will occur for that individual in the future.

Randomization

Randomization will occur using Epic EHR's randomization feature, built into the EHR.

Primary Outcome/Endpoint

Incidence of HA-VTE, VTE associated with healthcare encounter. We will exclude VTE present on admission (VTE-POA) as a study outcome.

Secondary Outcomes/Endpoints

Secondary trial outcomes include include process metrics – Length of Stay, Readmission Rates – and safety metrics – bleeding events.
Length of Stay is measured as days between admission and discharge.
Readmission Rates will be rates of readmission within 30-days of discharge for trial encounters related to VTE. Bleeding events are a safety risk from anticoagulation and DVT/VTE prophylaxis and will be monitored during the trial.

Standard of Care – DVT/VTE Prophylaxis

For patients admitted to an Adult Medicine Service, the order set prompts the following calculation of the Padua risk tool for VTE prognostication:

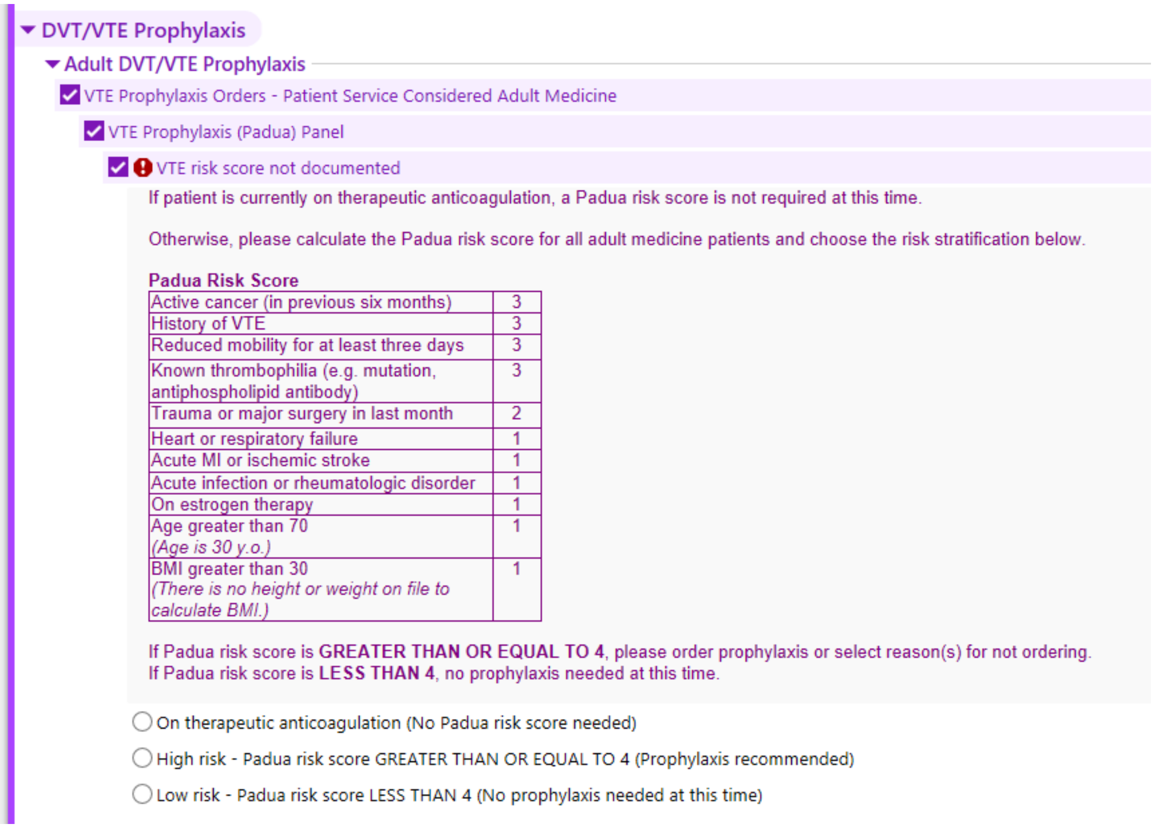

DVT/VTE Prophylaxis

Adult DVT/VTE Prophylaxis

☑ VTE Prophylaxis Orders - Patient Service Considered Adult Medicine

☑ VTE Prophylaxis (Padua) Panel

☑ VTE risk score not documented

If patient is currently on therapeutic anticoagulation, a Padua risk score is not required at this time.

Otherwise, please calculate the Padua risk score for all adult medicine patients and choose the risk stratification below.

Padua Risk Score

Active cancer (in previous six months)	3
History of VTE	3
Reduced mobility for at least three days	3
Known thrombophilia (e.g. mutation, antiphospholipid antibody)	3
Trauma or major surgery in last month	2
Heart or respiratory failure	1
Acute MI or ischemic stroke	1
Acute infection or rheumatologic disorder	1
On estrogen therapy	1
Age greater than 70 *(Age is 30 y.o.)*	1
BMI greater than 30 *(There is no height or weight on file to calculate BMI.)*	1

If Padua risk score is **GREATER THAN OR EQUAL TO 4**, please order prophylaxis or select reason(s) for not ordering.
If Padua risk score is **LESS THAN 4**, no prophylaxis needed at this time.

○ On therapeutic anticoagulation (No Padua risk score needed)

○ High risk - Padua risk score GREATER THAN OR EQUAL TO 4 (Prophylaxis recommended)

○ Low risk - Padua risk score LESS THAN 4 (No prophylaxis needed at this time)

For patients not currently listed as admitted to Adult Medicine, admitting physicians are prompted to indicate if patients will be admitted to Adult Medicine, the service using the DVT/VTE prophylaxis order set:

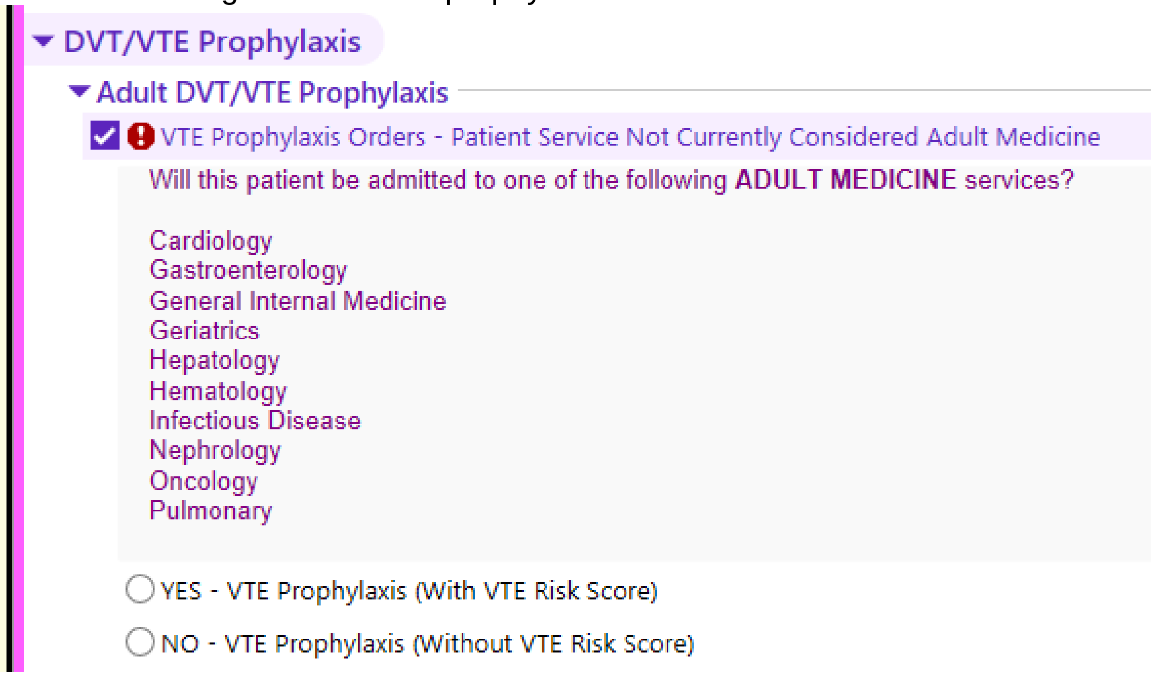

DVT/VTE Prophylaxis

Adult DVT/VTE Prophylaxis

☑ VTE Prophylaxis Orders - Patient Service Not Currently Considered Adult Medicine

Will this patient be admitted to one of the following **ADULT MEDICINE** services?

Cardiology
Gastroenterology
General Internal Medicine
Geriatrics
Hepatology
Hematology
Infectious Disease
Nephrology
Oncology
Pulmonary

○ YES - VTE Prophylaxis (With VTE Risk Score)

○ NO - VTE Prophylaxis (Without VTE Risk Score)

Selecting Yes prompts the Padua risk tool as above.

If the admitting physician selects “High Risk” on the Padua, they are prompted to select prophylaxis:

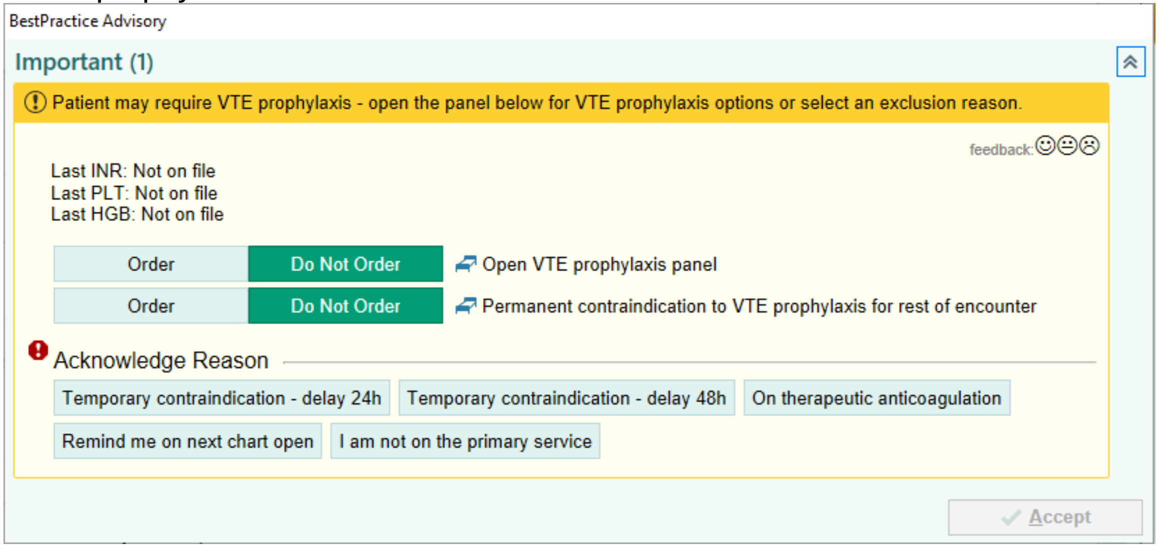

If they indicate a permanent contraindication “Order”, they are prompted to select the reason:

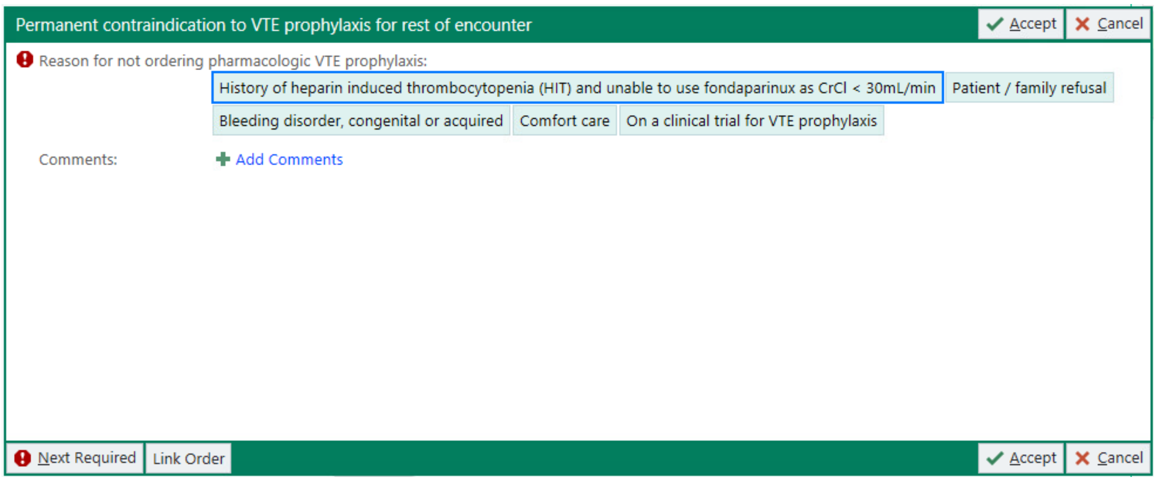

Intervention

The CDS intervention will include EHR "nudges" in the form of Best Practice Advisories targeting those encounters on which 1) VTE-AI risk is above threshold (defined below), 2) no active DVT prophylaxis pharmacologic order is present, 3) no contraindication has been documented in the current admission order sets above. The schematic below demonstrates the implementation.

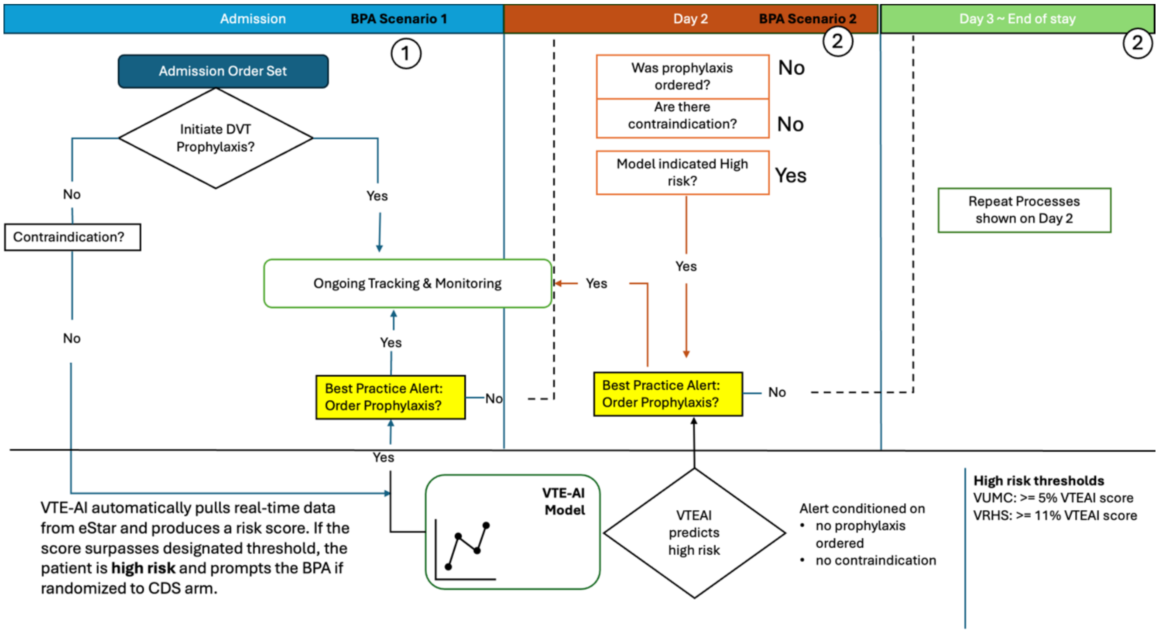

The CDS will prompt on order sign for admissions order sets (current state) and on Chart Review the subsequent morning for each day of an inpatient encounter. The CDS BPA is shown here and prompts consideration of pharmacologic prophylaxis and opportunities to take alternate actions including disagreeing with the alert, entering contraindications, or indicating the patient is already on acceptable prophylaxis.

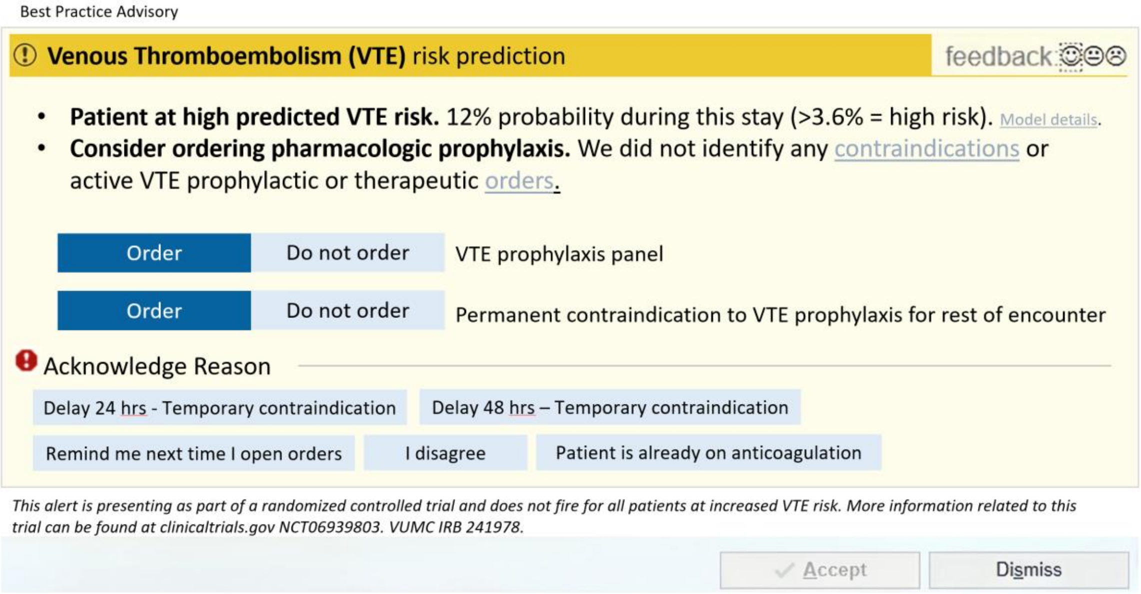

Discontinuing Interventions

The CDS will be discontinued by acting on any of the above buttons in the BPA, the Recommended or Alternate Actions.

Adherence to Intervention Protocols

Adherence to the primary intervention will be measured through direct interaction with the study decision support tools. We will also assess orders for all patients randomized in the trial to verify whether prophylaxis orders were made as well as data from admitting orders and existing BPAs related to VTE prophylaxis.

Relevant concomitant care and interventions
Decision to act on study CDS is left to practitioners in study settings. Any concomitant care is permitted at any point in the trial and during study encounters.

Participant Timeline
Randomization, interventions, and assessments all occur within each study encounter with no follow-up or additional interventions for either study participants (practitioners) or patients.

Sample Size
We hypothesize CDS will reduce incidence of HAVTE in those i) predicted at high risk by VTEAI and ii) without evidence of pharmacologic prophylaxis in half, from baseline 4.3% incidence (562/12,946 events) to 2.2% incidence, which will require 2,236 encounters. Sample size calculation indicates at least 1,118 patient encounters are needed in each arm to achieve 80% power with 5% probability of type I error. Using historical data from 2023-2024, we see encounter counts of 150-230 encounters per month meeting these criteria. We will therefore conduct the RCT for one (1) year to meet required sample size.

Recruitment
Because of the pragmatic nature of this RCT, enrollment/randomization occurs at the start of each encounter, when the VTE-AI risk score is calculated. No additional recruitment will be conducted.

Post-Implementation Evaluation
We will analyze the BPA utilization data routinely collected within the Epic EHR to identify providers who encountered the BPA in regular patient care. These data include logs of BPA views and actions taken as well as orders written via the BPA and linked order sets.

7.0 Risks
This protocol presents minimal risks to participants. The risk model might identify risk in those not at high risk of HA-VTE, leading to pharmacologic prophylaxis that might not otherwise have been ordered. Adverse events include potential increased bleeding risk if prophylaxis is ordered from the CDS prompt that would not otherwise have been ordered. Bleeding from VTE prophylaxis in general is a rare event. The risk model is not perfect and might not identify risk in those who are at risk – it is not intended to preempt providers prophylaxing any patient (or all patients) for HA-VTE if they choose to do so.

8.0 Reporting of Adverse Events or Unanticipated Problems involving Risk to Participants or Others

Harms

The PIs are responsible for monitoring study data, assuring protocol compliance, and conducting safety reviews. Because of the pragmatic nature of the study interventions (CDS decision aids), adverse events or other problems are anticipated to be rare. In the unlikely event that such events occur, unanticipated problems involving risks to subjects or others that are i) serious, ii) unanticipated, and iii) possibly or definitely related to research procedures will be reported immediately to the PI, and in writing within 7 days to the VUMC IRB. The PI will apprise fellow investigators and study personnel of such adverse events that occur during the conduct of this research project through regular study meetings. Continuing review reports will be submitted annually to the IRB summarizing study progress, adverse events, complaints about the research or withdrawals, and any protocol violations.

We will follow the VUMC Human Research Protections Program (VHRPP) guidelines that require investigators to promptly notify the IRB within 7 days of the occurrence when such unexpected Adverse Events (AEs) occur. These are events that could possibly be related to the intervention, or occur more frequently or are more severe than anticipated. VHRPP requires that any AE that is unexpected and related or possibly related to the research be reported. Adverse events not meeting this definition will be reported at the time of continuing review.

Unanticipated problems involving risks to subjects or others will be reported immediately, within twenty-four (24) hours of their discovery to the PI, and in writing within 7 days to the IRB. The PI will apprise fellow investigators and study personnel of all AEs that occur during the conduct of this research project through regular study meetings. Annual reports will be submitted by the study coordinator to the responsible IRBs summarizing study progress, AEs, complaints about the research, and any protocol violations.

Auditing

Study data will be analyzed by the PI and study team every three months for 1) continued validity of VTE-AI to prognosticate HA-VTE; 2) proper functioning of the VTE-AI model [e.g., ensuring all predictors continue to be calculated appropriately for patients]; 3) rates of HA-VTE and bleeding events. Because this study necessitates close collaboration with providers receiving BPAs, the study team will have monthly small-group virtual meetings with providers in study sites at VUMC to assess for problems or perceived AEs related to the study protocol, though none are anticipated.

9.0 Study Withdrawal/Discontinuation

Providers may dismiss the BPA and not act on its recommendations at any time. No other intervention is planned. Patients would be unaware of the providers’

decisions in that case unless the provider chooses independently to discuss the BPA with them.

10.0 Statistical Considerations

The study team determined the following power analysis.

We hypothesize CDS will reduce incidence of HAVTE in those i) predicted at high risk by VTEAI and ii) without evidence of pharmacologic prophylaxis in half, from baseline 4.3% incidence (562/12,946 events) to 2.2% incidence, which will require 2,236 encounters. Sample size calculation indicates at least 1,118 patient encounters are needed in each arm to achieve 80% power with 5% probability of type I error. Using historical data from 2023-2024, we see encounter counts of 150-230 encounters per month meeting these criteria. We will therefore conduct the RCT for one (1) year to meet required sample size.

11.0 Privacy/Confidentiality Issues

All study experiments occur solely in the context of already-scheduled clinical care and among patients and providers already intending to interact for purposes of healthcare delivery. No data outside that patient's health record are used in the calculation of VTE-AI or in study conduct. Because these interactions occur solely in routine healthcare encounters, the same confidentiality rules apply as in standard of care.

Study data are secured on servers in the VUMC data center or in VUMC Health Information Technology production systems only. These systems are secured via personal, physical, and technical controls including their invisibility to networks outside the VUMC firewall. User credentials are maintained by the VUMC Active Directory and Information Technology and are necessary to access any study data. Only those credentialed and on an approved IRB will have permissions to access study data on these servers. No paper records will be generated nor recorded in this study. All data collection will be digital and will leverage operational clinical systems and EHRs in their generation. No new data collection mechanisms will be required and therefore no new vulnerabilities generated.

12.0 Follow-up and Record Retention

The study will last six months.

As per IRB policy VI.B, all study data will be maintained for six (6) years from the date of the last use of study Protected Health Information (PHI) - at the conclusion of the trial period to identify patients in need of outcome data collection (which is based in EHR queries only).

13.0 Ethics and Dissemination

Institutional Review Board/Ethics Review Board Approval

VUMC IRB Approval pending.

Protocol Amendments

Changes to trial protocols including eligibility criteria, outcomes, or analyses will be communicated to the governing IRB, updated in national registration (ClinicalTrials.gov), clinical trial site leadership and participating clinicians within one calendar day.

Waiver of Consent – in-clinic preventive CDS

Waiver of consent will be pursued for the clinical intervention CDS. This study aims to measure the effectiveness of a risk model-driven BPA on provider behavior. Multiple factors contribute to impracticability to obtain consent. Consenting only those identified at highest risk would itself introduce selection bias and might alter behavior of staff/providers aware that consent had taken place in a manner that would alter study results. Informed consent at the start of the visit disrupts that intended workflow and might bias decision-making. Waiver prevents that issue from i) disrupting care for those patients and ii) unduly affecting the study itself.

Waiver of Consent – minimal risk

This study does not involve an investigational intervention given or administered directly to patients. This study poses no greater than minimal risk to study participants. VUMC has become a leader in pragmatic, minimal risk clinical trial designs like this one and we have engaged the Learning Health System for high-level guidance in study design. Our study team benefits greatly from the experiences and expertise of our colleagues who have completed similar studies under waiver of informed consent. The potential benefit of this study would come through better provision of screening to those at risk who would not otherwise be screened in current state. Waiver of consent will have no impact on privacy for patients or providers because the CDS will be applied only in routine encounters already scheduled for unrelated reasons, and no data from this study exist outside the bounds of the individuals' EHRs.

Confidentiality

Data will be collected from the EHR and stored securely in the VUMC Data Center on servers owned by the study team for later analysis. These systems are designed to securely store Protected Health Information and include encrypted storage, multifactor authentication, and both physical (locked doors, secure facility) and technical (active directory user authentication) controls. Protected Health Information, while necessary to conduct study analyses, will never be exported from these servers or shared.

Data to be collected to calculate VTE-AI include type of admission; heart rate; diagnostic codes (for clinical comorbidities); central line placement data; laboratory data including basic metabolic panel and c-reactive protein.

Declaration of Interests

No conflicts of interest exist, financial or otherwise, for any members of the study team relevant to this research.

Access to data

Two study team members have access to raw EHR data needed to conduct study analyses: Colin Walsh (PI), Michael Ripperger (developer/analyst). Only members of HealthIT with Physician Builder credentials necessary to design and implement the trial CDS might access EHR data within our vendor EHR: e.g., Dan Albert (HealthIT).

Ancillary and Post-trial care

Not applicable to this study.

Dissemination Policy

Our team will disseminate trial findings through peer-reviewed journals and national conferences including biomedical informatics, internal medicine, and hematology. Where possible, the team will preprint our manuscripts at the start of peer review for more transparent dissemination of our results. We will pursue open access publication where available to maximize dissemination.

No professional writers will be employed in dissemination.

Because of the sensitive nature of the PHI needed to conduct this trial, public access to participant-level data will not be possible. Statistical code and the full protocol for the study (this document) will be disseminated with peer-reviewed publication as appropriate.

14.0 Appendices

Informed consent materials

Waiver of consent, as above

Biological Specimens

Not applicable
