# Supplementary material for: AI-Driven Clinical Decision Support to Reduce Hospital-Acquired Venous Thromboembolism: A Trial Protocol
Source: JAMA Netw Open. 2025 Oct 3;8(10):e2535137. doi: 10.1001/jamanetworkopen.2025.35137 (PMC12495493; doi:10.1001/jamanetworkopen.2025.35137)
Supplement: Supplement 2. — eAppendix. [file jamanetwopen-e2535137-s002.pdf]

Supplemental Online Content

Walsh CG, Long Y, Novak LL, et al. AI-driven clinical decision support to reduce hospital-acquired venous thromboembolism: a trial protocol. *JAMA Netw Open*. 2025;8(10):e2535137. doi:10.1001/jamanetworkopen.2025.35137

eAppendix

This supplemental material has been provided by the authors to give readers additional information about their work.

© 2025 Walsh CG et al. *JAMA Network Open*.

eAppendix

Permanent Contraindications:

History of heparin induced thrombocytopenia (HIT) and unable to use fondaparinux as CrCl < 30mL/min
Patient / family refusal
Comfort care

On a clinical trial for VTE prophylaxis

Bleeding disorder, congenital or acquired

Temporary Contraindications the workflow

Contraindicated

On therapeutic anticoagulation

Other

Procedure planned

Bleeding or high risk for bleeding

Low platelets <50

TPA within previous 24 hours

Epidural catheter in place or planned

Excluded from other criteria:

Platelet count <30

Has a bleeding disorder FYI flag

On therapeutic anticoagulation

Blood factor order

Reversal agent for direct thrombin inhibitor ordered

Med admin within 24 hours for warfarin, DOAC, blood factors, anticoagulant reversal agent, enoxaparin, fondaparinux, argatroban, bivalirudin

List of Medications counting as therapeutic anticoagulation:

Pharmaceutical subclass:	Direct Factor Xa Inhibitors
Simple generic med:	ABCIXIMAB [156668]<br>ARGATROBAN [157858]<br>ARGATROBAN IN 0.9 % SOD CHLOR [160494]<br>ARGATROBAN IN NACL,ISO-OSMOTIC [160634]<br>BIVALIRUDIN [157883]

© 2025 Walsh CG et al. *JAMA Network Open*.

BIVALIRUDIN/0.9 % SODIUM CHLOR [191892]
DALTEPARIN SODIUM,PORCINE [156403]
ENOXAPARIN SODIUM [156456]
FONDAPARINUX SODIUM [158131]
WARFARIN SODIUM [155150]

Records

ALTEPLASE (TPA) BOLUS FROM INFUSION [501029]
ALTEPLASE 0.5 MG/ML IN 20 ML NS BOLUS [555637]
ALTEPLASE 10 MG IN 100 ML NS INFUSION FOR PROSTHETIC VALVE THROMBOSIS [555635]
ALTEPLASE 10 MG IN 1000 ML NS 0.01 MG/ML INFUSION (VASCULAR OCCLUSION) [551339]
ALTEPLASE 10 MG IN 250 ML NS 0.04 MG/ML INFUSION (VASCULAR OCCLUSION) [551337]
ALTEPLASE 10 MG IN 500 ML NS 0.02 MG/ML INFUSION (VASCULAR OCCLUSION) [551338]
ALTEPLASE 100 MG INTRAVENOUS SOLUTION [9002]
ALTEPLASE 15 MG IN 1000 ML NS 0.015 MG/ML INFUSION (VASCULAR OCCLUSION) [551341]
ALTEPLASE 15 MG IN 500 ML NS 0.03 MG/ML INFUSION (VASCULAR OCCLUSION) [551340]
ALTEPLASE 20 MG IN 1000 ML NS 0.02 MG/ML INFUSION (VASCULAR OCCLUSION) [551342]
ALTEPLASE 5 MG IN 1000 ML NS 0.005 MG/ML INFUSION (VASCULAR OCCLUSION) [551336]
ALTEPLASE 5 MG IN 250 ML NS 0.02 MG/ML INFUSION (VASCULAR OCCLUSION) [551334]
ALTEPLASE 5 MG IN 500 ML NS 0.01 MG/ML INFUSION (VASCULAR OCCLUSION) [551335]
ALTEPLASE 50 MG INTRAVENOUS SOLUTION [9003]
ALTEPLASE FOR EKOS CATHETER BOLUS [551159]
ALTEPLASE IN 50 ML D5W FOR FIBRIN SHEATH (PEDS) [552062]
ALTEPLASE IN NORMAL SALINE 100 ML [551149]
ALTEPLASE IN NORMAL SALINE 50 ML [551148]
ALTEPLASE IN NS 0.1 MG/ML FOR CATHETER DIRECTED TPA INJECTION (PEDS) [522041]
ALTEPLASE IN NS INFUSION [551145]
ALTEPLASE IN SWFI 1 MG/ML 100 ML [400650]
ALTEPLASE IN SWFI 1 MG/ML FOR HIGH DOSE [551155]
ALTEPLASE INFUSION - CATHFLO [400344]
ALTEPLASE INFUSION (RADIOLOGY) [400236]
ALTEPLASE INFUSION FOR EKOS CATHETER [551158]
ALTEPLASE INFUSION FOR FIBRIN SHEATH [551151]

© 2025 Walsh CG et al. *JAMA Network Open*.

ALTEPLASE INFUSION IN NS 100 ML [551157]
ALTEPLASE INFUSION IN NS 50 ML [551156]
ALTEPLASE 1 MG/ML INTRATHECAL SOLUTION [400078]
ALTEPLASE IV BOLUS [400077]
ALTEPLASE LOW DOSE INFUSION IN 1000 ML NS (PED) [552063]
ALTEPLASE LOW DOSE INFUSION IN 20 ML NS (PED) [555675]
ALTEPLASE LOW DOSE INFUSION IN 50 ML NS (PED) [551154]
HEPARIN (PORCINE) (PF) 1,000 UNIT/500 ML IN 0.9 % SODIUM CHLORIDE IV [15847]
HEPARIN (PORCINE) (PF) 2,000 UNIT/1,000 ML IN 0.9 % SODIUM CHLORIDE IV [127120]
HEPARIN (PORCINE) 1,000 UNIT/ML INJECTION SOLUTION [10176]
HEPARIN (PORCINE) 10,000 UNIT/ML INJECTION SOLUTION [10177]
ECMO HEPARIN (ADULT) FOR CANNULATION 10,000 UNITS/10 ML (1,000 UNITS/ML) SOLUTION [505150]
HEPARIN (PORCINE) 20,000 UNIT/ML INJECTION SOLUTION [10178]
HEPARIN (PORCINE) 25,000 UNIT/250 ML (100 UNIT/ML) IN DEXTROSE 5 % IV [24329]
HEPARIN (PORCINE) 25,000 UNIT/250 ML IN 0.45 % SODIUM CHLORIDE IV SOLN [15849]
HEPARIN (PORCINE) 5,000 UNIT/ML INJECTION SOLUTION [10181]
HEPARIN (PORCINE) IV BOLUS (FROM PHARMACY) [551173]
HEPARIN (PORCINE) IV BOLUS FROM INFUSION [501000]
HEPARIN 15000 UNITS/15 ML SYRINGE FOR CRRT [5512069]
HEPARIN 2,000 UNITS IN 1,000 ML NS (2 UNITS/ML) FOR CRRT (BAG) [552049]
HEPARIN 500 UNITS/0.5 ML SYRINGE FOR CRRT PRIMING CIRCUIT [551467]
HEPARIN BOLUS FROM CRRT SYRINGE/BAG [501212]
HEPARIN FOR CRRT SYRINGE [501211]
HEPARIN FOR DCD 30,000 UNITS/6 ML (OR) [505088]
HEPARIN INFUSION 40 UNITS/ML IN 1/2 NS 50 ML [400401]
HEPARIN INFUSION 40 UNITS/ML IN D5W 50 ML [551304]
HEPARIN INFUSION 5000 UNITS/1000 ML NS (5 UNITS/ML) [505012]
HEPARIN INFUSION FOR IMPELLA IN D5W [555066]
HEPARIN INFUSION FOR IMPELLA PUMP IN D20W [555067]
HEPARIN INFUSION IN NS [400402]
HEPARIN IVPB (FOR APHERESIS) [400667]

© 2025 Walsh CG et al. *JAMA Network Open*.

HEPARIN 10,000 UNITS/500 ML NS FOR PHOTOPHERESIS PROCEDURE [507016]
HEPARIN 5,000 UNITS/500 ML NS FOR PHOTOPHERESIS PROCEDURE [5115005]
HEPARIN PRIMING SOLUTION (FOR APHERESIS) [400668]
HEPARIN SYRINGE 1000 UNITS/1 ML [5912071]
HEPARIN, PORCINE (PF) 5,000 UNIT/0.5 ML INJECTION SYRINGE [119245]
ALTEPLASE IN 50 ML NS FOR FIBRIN SHEATH (PEDS) [555758]
ALTEPLASE LOW DOSE INFUSION IN 500 ML NS (PED) [555765]
TPA
Active warfarin consult

List of Medications Counting as Prophylaxis

HEPARIN, PORCINE (PF) 5,000 UNIT/0.5 ML INJECTION SYRINGE [119245]
HEPARIN (PORCINE) 5,000 UNIT/ML INJECTION SOLUTION [10181]
HEPARIN (PORCINE) 20,000 UNIT/ML INJECTION SOLUTION [10178]
ENOXAPARIN 30 MG/0.3 ML SUBCUTANEOUS SYRINGE [105899]
ENOXAPARIN 40 MG/0.4 ML SUBCUTANEOUS SYRINGE [105900]
ENOXAPARIN 300 MG/3 ML SUBCUTANEOUS SOLUTION [105940]
FONDAPARINUX 2.5 MG/0.5 ML SUBCUTANEOUS SOLUTION SYRINGE [32215]
APIXABAN 2.5 MG TABLET [119040]
RIVAROXABAN 10 MG TABLET [110250]
ENOXAPARIN 300 MG/3 ML SUBCUTANEOUS SOLUTION (DISPENSED IN INSULIN SYRINGE) (PEDS) [505456]
ENOXAPARIN 300 MG/3 ML SUBCUTANEOUS SOLUTION (DISPENSED IN SPLIT INSULIN SYRINGE) (PEDS) [505526]
HEPARIN, PORCINE (PF) 5,000 UNIT/0.5 ML SUBQ / INJ SYRINGE (WRAPPED RECORD) [5018138]

© 2025 Walsh CG et al. *JAMA Network Open*.
