# Supplementary material for: AI-Driven Clinical Decision Support to Reduce Hospital-Acquired Venous Thromboembolism: A Trial Protocol
Source: JAMA Netw Open. 2025 Oct 3;8(10):e2535137. doi: 10.1001/jamanetworkopen.2025.35137 (PMC12495493; doi:10.1001/jamanetworkopen.2025.35137)
Supplement: Supplement 3. — Data Sharing Statement [file jamanetwopen-e2535137-s003.pdf]

Data Sharing Statement

Walsh. AI-Driven Clinical Decision Support to Reduce Hospital-Acquired Venous Thromboembolism. *JAMA Netw Open*. Published October 03, 2025. doi:10.1001/jamanetworkopen.2025.35137

Data
Data available: No

Additional Information
Explanation for why data not available: Protected Health Information
